# Supplementary material for: Hi-D: nanoscale mapping of nuclear dynamics in single living cells
Source: Genome Biol. 2020 Apr 20;21:95. doi: 10.1186/s13059-020-02002-6 (PMC7168861; doi:10.1186/s13059-020-02002-6)
Supplement: Supplementary file 3 — Review history. [file 13059_2020_2002_MOESM3_ESM.docx]

**First round**

**Reviewer 1**

**Are the methods appropriate to the aims of the study, are they well described, and are necessary controls included?**

This paper is essentially a method development paper. Some basic biological experiments were done (depletion and addition of serum to alter transcription activities). But control is not sufficient (detailed in the comments) and there is also lacking other experimental approach to further support their conclusion.

**Are the conclusions adequately supported by the data shown?**

No. The authors reported a relatively counter-intuitive conclusion that the significantly slower diffusion rate of the cell nucleus for more de-compacted chromatin upon active transcription. Then the authors concluded the decoupled chromatin compaction and motility. However, their data also showed that the more compacted region within the nucleus (heterochromatin) exhibited slower diffusion rate. What they observed may be true, but given the inconsistency in their data, they should provide additional experimental support to justify their conclusion.

**Are sufficient details provided to allow replication and comparison with related analyses that may have been performed?**

Yes

**Does the manuscript adhere to the journal's guidelines on Minimum Standards Reporting Checklist?**

The number of cells (n = 13) was reported. However, the authors did not mention how many times the experiments were repeated. The simple statistical analysis was performed to justify the statistical significance. However, there was no power analysis done.

**Does the method perform better than existing methods (as demonstrated by direct comparison with available methods)?**

Yes

**Is the method likely to be of broad utility? Is any software component easy to install and use?**

The method may have some utility in the field of microscopic imaging to study chromatin biology. The software is not made publically available.

**Is the paper of broad interest to others in the field, or of outstanding interest to a broad audience of biologists**

No

This manuscript presents a method to quantitatively map DNA dynamics in the entire nucleus based on analyzing a series of consecutive fluorescence images. This paper is based on the recently published work from the same authors (H. A. Shaban, R. Barth, and K. Bystricky, "Formation of correlated chromatin domains at nanoscale dynamic resolution during transcription," Nucleic Acids Res, 46(13), e77 (2018)). In the previous work, the authors used the spatial and temporal image correlation to calculate optical flow maps and extracted several parameters to infer chromatin dynamics, such as correlation length and smoothness parameter that are indicative of directional correlation of ﬂow fields. This work extends the previous approach by including additional analysis to convert the flow fields to MSDs. As MSD analysis based on single-particle tracking has previously used to characterize chromatin dynamics into confined, random and anomalous diffusion, the authors extracted chromatin diffusion coefficient using Bayesian inference method.

Overall, although extraction of MSD parameters from the optical flow images is useful and is a nice extension of the previous approach, the reviewer felt that this paper does not go very far extending the previous approach. Importantly, the authors did not truly demonstrate the utility of these parameters by extensively studying a biologically significant biological problem. In the biological experiments, the authors used the similar sets of data (stimulated and quiescent cells with and without serum) as the previous work, which lacks in-depth investigation of an important biological question. Since this method is untested in biological setting, additional supporting biological experiments are needed to justify the claim of the observed decoupled chromatin compaction and mobility.

My main comments are listed below.

1. What is the biological basis for the reduced diffusion for stimulated cases? It is a little counter intuitive. For serum stimulation, the transcription is highly active and chromatin movement is expected to be faster. The authors' previous paper also suggested compaction decompaction upon serum stimulation, where faster chromatin movement is expected. Also, the authors showed in this work that more open euchromatin shows higher diffusion coefficient. This is inconsistent from the observation here where the diffusion coefficient upon serum stimulation is significantly reduced. The authors claimed that that is due to decoupled chromatin compaction and mobility. However, to justify this point, additional supporting experiments are needed to validate this point. Using an uncommon analysis to derive this conclusion without additional justification may lead to wrong conclusion.

2. The authors compared two extreme cases (serum starvation vs. serum stimulation). It is important to include a control group (without any treatment).

3. The authors classified different diffusion modes by assuming each region only presents one diffusion model. What if there are mixed cases where a specific region has mixed anomalous and confined diffusion?

4. Did the authors change the temporal resolution (e.g., 10-20 fps) and perform the same analysis? Do the same results hold? As the calculation of diffusion coefficient is based on statistical average, small change of temporal resolution should not dramatically change the results. If there is a significant variation, that will suggest the diffusion mode selected by Bayesian inference may not be accurate and the chromatin dynamics may contain some mixed modes as mentioned in the above question.

5. How does the authors account for the photobleaching effect in the model? As the image analysis is based on the spatial/temporal correlation of image intensity, the photobleaching effect may affect the result?

6. The classification of euchromatin vs. heterochromatin is simply based on their diffusion-based classification without biological validation. The authors should at least validate their classification with well-established heterochromatin and euchromatin markers.

7. How do the extracted parameters depend on the SNR of the images?

**Reviewer 2**

**Are the methods appropriate to the aims of the study, are they well described, and are necessary controls included?**

No. Direct comparison with other experimental methods is essential. A more detailed description of approaches is required and controls are missing. See response to authors below

**Are the conclusions adequately supported by the data shown?**

No. Though the method is potantially interesting, it has not be validated through comparison with other experimental methods

**Are sufficient details provided to allow replication and comparison with related analyses that may have been performed?**

No, experimental procedures lack details, see below

**Does the manuscript adhere to the journal's guidelines on Minimum Standards Reporting Checklist?**

Statistics are missing in a number of figures

**Does the method perform better than existing methods (as demonstrated by direct comparison with available methods)?**

No: maybe, but not shown - should be shown

**Is the method likely to be of broad utility? Is any software component easy to install and use?**

A method which would allow estimations of chromatin dynamics from analysing a simple time-series of DNA stained cells imaged for a few minutes in a conventional microscope would be of broad interest. The method has however been recently described by the same authors in NAR, 2018 (their reference 19) and a number of similar conlusions to the ones drawn here have been discussed in that paper. Here they extend the method to infer diffusion parameters but it is unclear whether they do this successfully or what additional insight this brings. Most importantly, the method has not been properly validated, in either this or their earlier publication.

**Is the paper of broad interest to others in the field, or of outstanding interest to a broad audience of biologists**

Yes: It would be if proven to be methodologically sound

The manuscript by Shaban et al builds on a previous method recently reported by the same authors (Nucleic Acids Research 2018) on optical flow analysis of time-lapse images of DNA stained cells to infer movement of chromatin. Though analysing the dynamics of chromatin movement can indeed provide important information on cellular physiology and therefore methods to facilitate this would be of general interest, the present manuscript has problems at multiple levels, which make it unsuitable for publication.

Major points

1. First and foremost, the authors have not compared their method to previously available methods and previously analysed physiological situations, in order to validate their findings. They derive some numbers from their analysis (optical flow analysis followed by Bayesian inference of diffusion parameters, from which they derive numbers such as diffusion constant, anomalous exponent and drift velocity), but it is unclear whether these numbers are an accurate read-out of chromatin dynamics. They have used simulations to probe the suitability of their approach under certain assumptions, but simulations are not sufficient for validation - direct comparison with other experimental methods under different physiological situations is essential. A number of methods have previously been reported to track the movement of chromatin in live cells (for example track a small number of labelled loci, track photo-activated histones or clusters of loci such as cy3dUTP labelled foci) and used to analyse changes in chromatin dynamics under different physiological situations. The authors should directly compare their method to these experimental methods and show that the chromatin dynamics they infer through their approach are consistent with those inferred through other methods and that they can detect physiologically relevant changes in chromatin dynamics. Without such comparisons, their approach lacks validation and cannot be used to reach biologically relevant conclusions.

2. They do not describe in sufficient detail what analysis they have done to reach their conclusions. For example, it seems that figures 1 and 2 are based on the analysis of a single time-series from a single cell each. Have they analysed a large number of cells and how consistent are their findings? Statistics should be given. Are these cells grown in the presence of serum? In that case, different cells within the population will be in different cell cycle phases and chromatin dynamics are likely to be affected by cell cycle phase, for example by ongoing replication. Have they controlled for cell cycle phase? Live-cell markers are available which can allow them to group cells based on cell cycle phase.

3. In figures 3 and 4 authors aim to compare transcriptionally active and inactive cells. Again, the description of exactly how they have set up their experiments is rather cryptic but they seem to assume that cells grown for 24 hours in the absence of serum are transcriptionally inactive while cells re-fed with serum for 10 min are transcriptionally active. This is an over-simplification and the authors do not seem to have carried out any controls. At the very least, the authors cannot refer to these cells as transcriptionally active and inactive - they have not shown this is the case.

In conclusion, while a method to derive chromatin dynamics from a simple time-series of DNA stained cells would be very useful for the community, the authors need to validate their method by direct comparison to other available experimental methods, before they use the method to draw physiologically relevant conclusions. In addition, experiments performed must be described in more detail and cellular physiology better controlled, if physiological conclusions are to be drawn. Finally they should better describe which aspects of their manuscript offer novel insight as compared to their recent NAR manuscript.

**Reviewer 3**

**Are the methods appropriate to the aims of the study, are they well described, and are necessary controls included?**

The authors describe their approach, and include important controls like a simulated test of performance and crosslinked cells. The description of the approach and rationale could still be clarified in some places for the benefit of non biophysical scientists.

**Are the conclusions adequately supported by the data shown?**

The conclusions are stated carefully to avoid overinterpretation in most cases. I am somewhat concerned that the "3 populations" of mobility that arise from their fitting of distributions to their data may be too artificial: why can't there be a continuum of diffusion types rather than hard cutoffs between similar pixels?

**Are sufficient details provided to allow replication and comparison with related analyses that may have been performed?**

Yes.

**Does the manuscript adhere to the journal's guidelines on Minimum Standards Reporting Checklist?**

Yes, as far as I can tell.

**Does the method perform better than existing methods (as demonstrated by direct comparison with available methods)?**

No: I really would answer "Yes and No"--- The authors do a good job comparing the performance to Single Particle Tracking and showing where one outperforms the other. But, they do not provide a direct comparison to conclusions from their previous velocity and correlation calculations: these are not the same methods, though, so a comparison may be tricky.

**Is the method likely to be of broad utility? Is any software component easy to install and use?**

This method could in theory be used by many researchers interested in intra-nuclear dynamics. Unless I spent more time trying to implement the method, though, I cannot comment on how straightforward it is to implement. Further, I might personally use the more direct measurements from their NAR paper rather than the complex classifications of their diffusion types shown here, unless they can provide really direct evidence that the diffusion calculations provide more insight than velocity and correlation alone.

**Is the paper of broad interest to others in the field, or of outstanding interest to a broad audience of biologists**

Yes: It will be of interest to others in the chromatin dynamics field

**Response to reviewers**

**Reviewer 1**

*This manuscript presents a method to quantitatively map DNA dynamics in the entire nucleus based on analyzing a series of consecutive fluorescence images. This paper is based on the recently published work from the same authors (H. A. Shaban, R. Barth, and K. Bystricky, "Formation of correlated chromatin domains at nanoscale dynamic resolution during transcription," Nucleic Acids Res, 46(13), e77 (2018)). In the previous work, the authors used the spatial and temporal image correlation to calculate optical flow maps and extracted several parameters to infer chromatin dynamics, such as correlation length and smoothness parameter that are indicative of directional correlation of flow fields. This work extends the previous approach by including additional analysis to convert the flow fields to MSDs. As MSD analysis based on single-particle tracking has previously used to characterize chromatin dynamics into confined, random and anomalous diffusion, the authors extracted chromatin diffusion coefficient using Bayesian inference method.*

*Overall, although extraction of MSD parameters from the optical flow images is useful and is a nice extension of the previous approach, the reviewer felt that this paper does not go very far extending the previous approach. Importantly, the authors did not truly demonstrate the utility of these parameters by extensively studying a biologically significant biological problem. In the biological experiments, the authors used the similar sets of data (stimulated and quiescent cells with and without serum) as the previous work, which lacks in-depth investigation of an important biological question. Since this method is untested in biological setting, additional supporting biological experiments are needed to justify the claim of the observed decoupled chromatin compaction and mobility.*

*My main comments are listed below.*

*1. What is the biological basis for the reduced diffusion for stimulated cases? It is a little counter intuitive. For serum stimulation, the transcription is highly active and chromatin movement is expected to be faster. The authors' previous paper also suggested compaction decompaction upon serum stimulation, where faster chromatin movement is expected. Also, the authors showed in this work that more open euchromatin shows higher diffusion coefficient. This is inconsistent from the observation here where the diffusion coefficient upon serum stimulation is significantly reduced. The authors claimed that that is due to decoupled chromatin compaction and mobility. However, to justify this point, additional supporting experiments are needed to validate this point. Using an uncommon analysis to derive this conclusion without additional justification may lead to wrong conclusion.*

Growing cells in serum-free media has been used as a physiological state to inactive transcription for both chromatin and RNA polymerase II (RNA Pol II) imaging [1–4]. The removal of serum from the culture medium arrests cells in G0, where transcription is less activated due to decrease of RNA Pol II synthesis and mRNA degradation [2,5,6]. It also leads to loss of proteins that bind to DNA, which makes the DNA less constrained with higher diffusion constant. In contrast, serum induction activates the mitogen-activated protein kinase pathway and leads to rapid nuclear accumulation of phosphorylated kinases (predominantly extracellular signal-regulated kinase) [7]. This signalling activates the transcription by increasing the level of RNAPII-Ser5P, which leads to protein-DNA binding, and therefore, the motion is supposed to be more constrained, in comparison to serum-free condition. Furthermore, to examine the role of the transcriptional process in domain structure and dynamics, we treated cells with DRB which is a selective inhibitor of transcription elongation by RNA Pol II in eukaryotic cells. DRB treatment did not change chromatin dynamics, suggesting that, although RNA Pol II activity is not directly involved in domain maintenance [8].

To confirm our observation, we performed live cell imaging for RNA Pol II in the same cell line, U2OS, and also under the same conditions in in serum- starvation and stimulating. In addition, we test the effect of transcription elongation inhibitor, 5,6-Dichloro-1-β-Dribofuranosylbenzimidazole (DRB) on both DNA and RNA Pol II dynamics sing our Hi-D method.

We added the following sentences to the main text in order to explain the serum effect.

Growing cells in a serum-free medium arrests cell cycling in G0 in which transcription is globally inhibited; adding serum to the medium brings cells to normal growth conditions with transcriptional activity [1–4].

*2. The authors compared two extreme cases (serum starvation vs. serum stimulation). It is important to include a control group (without any treatment).*

We apologize for the poor writing of this part. We have edited the text and added additional experiments. Please check out the answer of the question 1, which explains the difference between the serum and no serum conditions. In our study, imaging cells grown in serum is the normal (control) state, and we compare it to starved cell (inactive transcribed state). Additionally, we treated cells with RNA Pol II elongation inhibitor 5,6-Dichloro-1-β-Dribofuranosylbenzimidazole (DRB). So now we have three conditions for imaging both DNA and RNA Pol II motion.

*3. The authors classified different diffusion modes by assuming each region only presents one diffusion model. What if there are mixed cases where a specific region has mixed anomalous and confined diffusion?*

Thank you for this important question, yes we agree that Bayesian classification is not sensitive enough to resolve between anomalous and confined diffusion. From a theoretical standpoint, values for small time lags are weighted more since the (co-) variance is small for small time lags. On this account, the behaviour of theoretical and experimental MSD curves for small time lags has a large impact on model selection and is therefore an indication for the preference of the model of confined motion against anomalous diffusion (Supplementary Figure 3). However, the modelling functions of confined and anomalous diffusion are similar for short time lags and deviate for long time lags (anomalous diffusion keeps rising, albeit slow, whereas confined motion reaches a plateau). We performed a study that provides a detailed view on the comparison of confined and anomalous diffusion in order to test the ability of the Bayesian classification routine used in this study to resolve confined and anomalous diffusion (Supplementary Note 3 in the original manuscript, Note 2 in the revised version). This study suggests that sub-diffusive behaviour observed for chromatin may be best described by anomalous diffusion rather than confined diffusion to prevent misclassification and misinterpretation. Then, we gave for Bayesian classifications five motion models (free diffusion (D), anomalous diffusion (DA), directed motion (V) or a combination (DV) or (DAV) and excluded the model of confinement. However, the Bayesian inference scheme was able to resolve mixed diffusion regions in the same pixel such as cases of free diffusion plus direct motion (DV) and anomalous diffusion plus directed motion (DAV).

*4. Did the authors change the temporal resolution (e.g., 10-20 fps) and perform the same analysis? Do the same results hold? As the calculation of diffusion coefficient is based on statistical average, small change of temporal resolution should not dramatically change the results. If there is a significant variation, that will suggest the diffusion mode selected by Bayesian inference may not be accurate and the chromatin dynamics may contain some mixed modes as mentioned in the above question.*

We performed a single cell analysis for different time acquisition, 100ms (10ps), 200ms (5ps) and 300ms (3.5ps) and checked the effect of change in temporal resolution on our calculations.

The results of this figure are mentioned in Fig. 2 of the revised version and, in more detail, in Supplementary Fig. 9.

*5. How does the authors account for the photobleaching effect in the model? As the image analysis is based on the spatial/temporal correlation of image intensity, the photobleaching effect may affect the result?*

We apologize for not mentioning the photobleaching effect. Photobleaching in our experimental images was negligible. However, even if photobleaching would be more prominent, Hi-D would deliver consistent results (within limits). The reason is that photobleaching would, in first place, only affect the flow field estimation by Optical Flow directly. All other quantities are derived from flow fields. Flow fields, however, are estimated **only** between subsequent frames within the time series, between which photobleaching can be safely neglected. However, photobleaching leads to a decrease of the signal-to-noise ratio (SNR) over time and thus, experimentalists adapting Hi-D for their research should be aware that the SNR does not drop below a critical value. An investigation of the effect of SNR on the parameter estimated derived by Hi-D is presented in Fig. 2 of the revised manuscript and implications of photobleaching are mentioned in the main text as follows:

To confirm the robustness of extracted parameter values with respect to varying levels of imaging noise, Hi-D was applied to imaged nuclei to which noise was artificially added. The signal-to-noise ratio (SNR) of the original nuclei were about 26 dB and the SNR was reduced down to 16 dB. The computed distribution of diffusion constants is consistent up to a lower limit of about 20 dB, below which the distribution is considerably biased towards larger values and broadens (Figure 2d, Supplementary Figure 8). Likewise, features of the spatial map of diffusion constants is equally conserved for SNR values as low as 20 dB, demonstrating the robustness of Hi-D for varying imaging noise (Supplementary Figure 8). In analogy to the robustness to varying SNR levels, Hi-D is thus robust to photobleaching effects (provided that SNR ≥ 20dB) since flow fields are only estimated between consecutive images, between which illumination changes due to photobleaching are usually negligible.

*6. The classification of euchromatin vs. heterochromatin is simply based on their diffusionbased classification without biological validation. The authors should at least validate their classification with well-established heterochromatin and euchromatin markers.*

In the first version, euchromatin and heterochromatin were spatially classified in two different cell lines (U2OS and MCF-7 cells) based on their signal intensity, as described in [9]. Here, we validated this classification and biological conclusion by using well-established heterochromatin and euchromatin markers in NIH3T3 cells expressing both GFP-HP1α and mCherry-H2A, respectively. We applied the same analysis which yielded the same conclusions.

We added the following sentences to the main text.

These findings were confirmed in NIH3T3 cells expressing both mCherry-H2A and GFPHP1α, two well-established markers for the eu- and heterochromatin (Supplementary Figure 12). We could not detect reduced dynamics of either of the two types of chromatin, but instead, Hi-D analysis hints towards previously proposed liquid phase-separation of HP1α [10].

*7. How do the extracted parameters depend on the SNR of the images?*

Yes, we did evaluate the effect of the SNR for our calculation.

We have added a new figure for method validation (Figure 2) including the effect of SNR on the derived parameters. A more detailed analysis is shown in Supplementary Fig. 8.

A new sentence was added to the main text as follows:

Furthermore, Hi-D was also shown to be robust to slight variations in the time resolution of acquired time series as long as the expected motion between frames is in the order of the pixel size of the imaging system used (Supplementary Figure 9).

We also added the filter used to treat images of varying SNR in the Methods section, which serves as a guide for future treatment of noisy images.

Images of varying noise levels were treated with a bilateral filter with half-size of the Gaussian bilateral filter window of 5 pixels, the spatial-domain standard deviation value was set to 5 pixels and the intensity-domain standard deviation was varied from 0.3 to 0.8 for decreasing levels of the signal-to-noise ratio from 26 dB to 16 dB.

**Reviewer 2**

The manuscript by Shaban et al builds on a previous method recently reported by the same authors (Nucleic Acids Research 2018) on optical flow analysis of time-lapse images of DNA stained cells to infer movement of chromatin. Though analysing the dynamics of chromatin movement can indeed provide important information on cellular physiology and therefore methods to facilitate this would be of general interest, the present manuscript has problems at multiple levels, which make it unsuitable for publication.

Major points.

*1. First and foremost, the authors have not compared their method to previously available methods and previously analysed physiological situations, in order to validate their findings. They derive some numbers from their analysis (optical flow analysis followed by Bayesian inference of diffusion parameters, from which they derive numbers such as diffusion constant, anomalous exponent and drift velocity), but it is unclear whether these numbers are an accurate read-out of chromatin dynamics. They have used simulations to probe the suitability of their approach under certain assumptions, but simulations are not sufficient for validation - direct comparison with other experimental methods under different physiological situations is essential. A number of methods have previously been reported to track the movement of chromatin in live cells (for example track a small number of labelled loci, track photo-activated histones or clusters of loci such as cy3dUTP labelled foci) and used to analyse changes in chromatin dynamics under different physiological situations. The authors should directly compare their method to these experimental methods and show that the chromatin dynamics they infer through their approach are consistent with those inferred through other methods and that they can detect physiologically relevant changes in chromatin dynamics. Without such comparisons, their approach lacks validation and cannot be used to reach biologically relevant conclusions.*

The novelty and power of our Hi-D approach is the capability to track bulk structures with sub-pixel information from any fluorescence images with no need for prior experiences in gene labelling and use of advanced microscopes, which other tracking methods cannot offer. To validate our method and prove the numbers out of the analysis are an accurate read-out of chromatin dynamics, we compared Hi-D to iMSD, a well-established method to reveal dynamics of fluorescently labelled proteins, based on intensity fluctuations. This method is using, as Hi-D, camera based imaging systems and does not require single loci labelling or specific particle tracking and is thus suitable to validate our approach. The results are summarized in Figure 2. Together with the existing comparison to SPT, this completes the validation of Hi-D using orthogonal approaches.

We added the following to the main text and supplementary note 4:

We further validated Hi-D against iMSD, a well-established method to extract dynamic information of dense molecules, based on the spatial correlation function of intensity fluctuations caused by diffusing molecules, which are recorded using camera-based systems [11]. iMSD is usually applied to a region of interest, over which average parameters for the region of interest are extracted. To compare the Hi-D and iMSD approaches, iMSD was applied to successive ROIs spanning an entire nucleus with large overlap, such that parameters were computed for each pixel separately. The resulting maps of diffusion constants showed that regions of high and low mobility were apparent in both methods (Figure 2e, yellow regions marked with red arrows), thus demonstrating qualitative agreement between the two conceptually different methods. iMSD could not compute parameters for ROIs at the nuclear periphery, at which diffusion is expected to be exceptionally low, and in regions of very high mobility (Figure 2e, right, surrounded by yellow-coloured pixels). Quantitatively, both methods yield diffusion constants in the same order of magnitude (Hi-D (1.6 ± 0.8) ⋅ 10−3 𝜇𝑚2/𝑠 , iMSD (2.2 ± 4.5) ⋅ 10−3 𝜇𝑚2/𝑠, mean ± standard deviation). However, we noticed a bias towards very small (< 5 ⋅ 10−4 𝜇𝑚2/𝑠), and the presence of a few large (> 5 ⋅ 10−3 𝜇𝑚2/𝑠) values, and a considerable spread of the distribution compared to values extracted by Hi-D (Figure 2f). Likewise, values of the anomalous exponent extracted by iMSD showed many spurious values around 𝛼 ~ 0 and 𝛼 ~ 2, while the distribution of values derived from iMSD is reasonable with respect to the magnitude of extracted parameters (0.25 < 𝛼 < 1.5, Figure 2g). We conclude that the estimation of dynamic parameters using Hi-D yields parameters which are qualitatively and quantitatively similar to an orthogonal approach, iMSD, and is thus an accurate read-out of chromatin dynamics. By making use of a Bayesian inference to select a suitable interpretive model for regression of MSD curves, Hi-D is advantageous to existing methods to yield conclusive and spurious-free parameter estimates within the entire nucleus simultaneously. It is to mention that iMSD is applied to ROIs spanning ~ 50 𝜇𝑚2 [11] (about 70 x 70 pixels in our data), while Hi-D uses only a 3 x 3 pixel neighbourhood to estimate the correct diffusion model and its parameters and thus offers greatly enhanced spatial specificity. Finally, we observed a ~ 35 speed-up in computational time of Hi-D compared to iMSD for a whole nucleus (roughly 500 𝜇𝑚2) with single-pixel resolution.

*2. They do not describe in sufficient detail what analysis they have done to reach their conclusions. For example, it seems that figures 1 and 2 are based on the analysis of a single time-series from a single cell each.*

Figure 1 explains the method structure and development showing only one cell, now we deleted any conclusion to avoid any over interpretation. Figure 2 (old version) is omitted and some plots have been moved to Figure 1 and one plot to Supplementary Figure 10. Figure 2 (new version) is dedicated to the method validation.

*Have they analysed a large number of cells and how consistent are their findings? Statistics should be given.*

Figure 4 and 5 are for large number of cells, and also statistical analysis was given*.*

*Are these cells grown in the presence of serum?,* Yes

*In that case, different cells within the population will be in different cell cycle phases and chromatin dynamics are likely to be affected by cell cycle phase, for example by ongoing replication. Have they controlled for cell cycle phase? Live-cell markers are available which can allow them to group cells based on cell cycle phase.*

Growing cells in a serum-free medium arrests cell cycling in G0 in which transcription is globally inhibited, adding serum to the medium bring cells to normal growth conditions and activates transcription. The time of serum supplement (10-20 minutes) is very short to initiating replication replication.

*3. In figures 3 and 4 authors aim to compare transcriptionally active and inactive cells. Again, the description of exactly how they have set up their experiments is rather cryptic but they seem to assume that cells grown for 24 hours in the absence of serum are transcriptionally inactive while cells re-fed with serum for 10 min are transcriptionally active. This is an over-simplification and the authors do not seem to have carried out any controls. At the very least, the authors cannot refer to these cells as transcriptionally active and inactive - they have not shown this is the case.*

We apologize for not being clear. This section was edited and more experimental details were included in the method section. Also, new experiments imaging RNA Pol II in cells treated or not with transcriptional elongation inhibitor, DRB, were added to evaluate the dynamic properties of both DNA and RNA Pol II during transcription.

*In conclusion, while a method to derive chromatin dynamics from a simple time-series of DNA stained cells would be very useful for the community, the authors need to validate their method by direct comparison to other available experimental methods, before they use the method to draw physiologically relevant conclusions. In addition, experiments performed must be described in more detail and cellular physiology better controlled, if physiological conclusions are to be drawn. Finally they should better describe which aspects of their manuscript offer novel insight as compared to their recent NAR manuscript.*

**Reviewer 3**

*This manuscript presents an extension of the authors previously published whole nucleus chromatin motion tracking approach which allows them to classify regions of the nucleus according to their diffusive parameters. The authors draw heavily on their previously published observations (increased motion in serum starvation) and previously published analysis methods, limiting their novelty somewhat, but they do well to push further beyond the basic observation to potential explanations and more specific quantitation of observations. The basic results are interesting, and the method in general is likely to lead to more interesting insights in the future. However, I have some concerns and suggestions for revision about the presentation of the material- how some aspects are presented or interpreted.*

*Comparison with previous study I would appreciate a more explicit description of how the approach to diffusion classification taken here revealed insights that were not apparent from their earlier description of velocity and correlated motion.*

*For example, it would be helpful to see how the interpretations about dynamics and heterochromatin change, if at all, when using the basic initial motion calculation as in the NAR paper- apart from inferred physical parameters, how does the basic motion of a pixel vary with its presence in heterochromatin/euchromatin?*

Thank you for the suggestion. We have analysed the flow magnitude as “raw” motion in euand heterochromatin, as well as the diffusion coefficient found in both chromatin types. The results are added to Figure 5 and show that not only populations do not show a trend to be associated with either chromatin type, but that also flow magnitude and diffusion constant are not affected by their location in euchromatin or heterochromatin.

*Issues with Figures:*

*Figure 1c, right, should indicate that this is "Drift Velocity" rather than making it appear as if this is the magnitude of the flow field vector for each pixel.*

Thank you. We changed the x-axis label to "Drift velocity”

*Figure 1c, centre, is confusing, because while pixels that have no "A" component of their classification are just white, there are also pixels with an apparent anomalous coefficient (greenish colour) very close to 1 (which would also be no anomalous component). How is this possible? It would be easier to visually judge which regions are truly anomalous if these anomalous coefficients very close to 1 were excluded (turned white) and then a white strip on the colorbar indicated that coefficients of 1 are shown in white.*

Indeed, pixels with anomalous exponent of 1 are shown in white as suggested, while any pixel with α ≠ 1 are shown in their respective colour (not white). The extent of “anomaly” may thus be judged by colour: anomalous diffusion with α close to 1 are greenish, while stronger anomaly is shown in blue.

*Serum starvation vs. stimulation If figure 2 is all for serum starved U2OS cells, this should be indicated at the beginning of the results portion describing this figure. Otherwise, a reader is left to infer this only after reaching the "serum stimulated" section.*

We apologize for the lack of clarity in this section. In the revised version, panels of the original Figure 2 are moved partially to Fig. 1 (deconvolved histograms by GMM) and to Supplementary Figure 10. The revised Fig. 2 is dedicated to the experimental validation of the Hi-D approach thus making the previous confusion about Figure 2 redundant.

*Interpretation of "3 populations"The authors provide a good extensive description of their mixture model EM approach to deconvolving the D and alpha distributions (Fig 2) into distinct populations. However, it seems a bit artificial to then colour these populations as completely distinct in the nucleus image when some pixels have an equal or nearly equal likelihood of coming from either population. Also, how can the authors be sure that the different populations derived under serum starved vs stimulated conditions actually mean the same thing in the two different ranges of values—ie, is it fair to label some as reclassified into a different group between conditions?*

Thank you for this important comment. Indeed, the colouring makes the distinction into three populations seem stricter than it is. As you mentioned correctly, two actually close values may fall in two different populations. In reality however, boundaries between populations are certainly smooth and the colouring employed here is an abstract stylization. For a complete view on transitions between regions of high and low mobility, we refer to maps which are shown in a heat-map with full dynamic range of parameter values (e.g. Fig. 1c). However, the colouring is a means of visualization and helps to spatially dissect where the populations are to be found within the nucleus.

We refer to the classification of parameters into sub-populations as a descriptive measure. At this stage, we cannot comment on possible underlying mechanisms forming these three populations and, as correctly pointed out, we do not know if the three populations mean the same thing in quiescent and stimulated cells. Nevertheless, the three populations are a relative measure compared to the nucleus as an entity (in the sense that pixels falling in one population are, within limits, different from pixels in the other populations). Therefore we find that it is suitable to describe that a pixel, previously classified in one population, becomes associated to another population upon serum stimulation (for instance). An example will demonstrate this more clearly: A pixel classified in the fast population in a quiescent cell indicates that chromatin shows higher mobility at this pixel than at others. If this pixel becomes re-classified into the intermediate or slow population upon serum stimulation, it means that, compared to the average dynamics observed within the whole nucleus, the previous high mobility is reduced to or below average. To clarify the use and interpretation of the three populations, we added the following to the discussion:

The use of three distinct populations does not reflect the real, likely fluent transitions between the populations, but emerged as a handy means of visualization and characterization of the highly complex and heterogeneous dynamic landscape of chromatin.

*Lack of clarity in long, complicated sentences with possible grammatical errors*

*This is a very long sentence that needs breaking up for clarity (page 8): "We find that heterochromatin exhibits enhanced spatial autocorrelation compared to euchromatin across all accessible time lags in line with our hypothesis and that spatial autocorrelation decreases with increasing time lag in serum-starved cells, while in serum-stimulated cells, autocorrelation is enhanced in the long-time limit (over 30 seconds)"*

*Similarly, there are number of places that need grammatical clarification or just conceptual clarification to make them easier to interpret. For example this sentence is hard to parse: "In conclusion, the combination of diffusion constant and anomalous exponent maps was complementary and provide an integrated view on chromatin organization and dynamics during genomic processes." Do you mean diffusion constant maps were complementary to anomalous exponent maps? Or these two together were complementary to something else? And the grammar should either be "were complementary and provide" or "was complementary and provides" (either single or plural consistently.*

Thank you for pointing this put. We improved our manuscript with respect to comprehensibility and grammatical correctness.

*Technical:*

*The authors' comparison of HiD and single particle tracking using simulated ground truth data is a nice demonstration of their method's utility.*

*Flat cells only?: Something that is true in both this study and the NAR paper is that, if I understand correctly, dynamics are only considered in 2D (not the Z direction). Will this method then only work for nuclei that are highly flattened in the Z direction, otherwise diffusion in Z would confound results, right? If a treatment increases the rounding up of a nucleus, everything would go out of the focal plane? How much does live cell motion and cytoskeleton dynamics affect how well you can accurately trust only 2D data?*

Yes, Hi-D in the version is developed for 2D analysis, and motion in Z is neglected. We agree with the reviewer that tracking of motion in spherical objects (although U2OS cells have a rather flat nucleus), in particular directed motion such as motion due to cytoskeleton dynamics effect could confound diffusion in Z. In general, this problem applies to all 2D tracking methods not only for our approach, and depends on how symmetric the motion is.

For isotropic motion, a 2D or 3D analysis will yield the same diffusion coefficient. Hence, the proper analysis of a 2D projection of a random trajectory is sufficient for determining the diffusion coefficient when the motion is isotropic. In time scale of our experiment (30 sec), we don’t expect a dramatic change in Z. We had seen this effect at time scale of minutes, where a deformation in the nuclear envelope was noticed (unpublished data). To confirm how accurate the motion in Z could affect the diffusion calculation in 2D, we need to develop our method for 3D imaging and compare 2D and 3D analysis in different cell shapes and different conditions, which is an ongoing project right now. At the current stage however, we have no reason to assume that motion in Z is in any sense special from motion in X and Ydirection (in other words, we can assume that motion is isotropic) and can thus safely derive diffusion constants and anomalous exponents observed in two dimensions.

*The authors point out that there is a rim of slow, free diffusion at the nuclear periphery. They hypothesize that this might come from nuclear confinement. It would be helpful for them to also discuss how they think lamina or nuclear pore tethering of chromatin might relate to this free diffusion coefficient. How can it be free diffusion if there is some tethering?*

Hi-D analysis revealed three fractions of DNA diffusing, the slowest mobility fraction is prominently located at the nuclear rim, is reminiscent of lamina associated domains (LADs). The MSD model selection for this fraction is chosen highly by free diffusion model, and partially by anomalous diffusion and diffusion with velocity models, therefore, the mobility of this rim does not follow one diffusion regime. Generally, LADs consist of two major classes: constitutive and facultative LADs. Both do interact with the nuclear lamina and nuclear sub compartments. Facultative LADs represent about half of all LADs, and are more gene-dense than constitutive LADs; these genes change their positions relatively to the nuclear lamina during nuclear processes [12]. Separation of these genes from nuclear lamina regularly occurs with gene activation while attachment to the nuclear lamina is normally associated by gene inactivation. One scenario might explain the dynamic behaviour of the rim: LADs can be formed in two regions, one is anchored to the nuclear lamina, while other regions may be tethered to nuclear structures that are free diffusive for transcription such as splicing factor speckles or transcription factories. Note that our compaction map shows that most of the rim is compact so one possibility could be that one or several genes within these chromatin regions are very compact and therefore likely move as a single bead (from polymer physics single beads in non-equilibrium state tend to move in free diffusive regime) [13]. Another hypothesis is that some LADs detach from the nuclear lamina irregularly and could be converted transcriptionally active when accompanied with a permissive compartment [14– 16]. These hypotheses indicate the elasticity and dynamics of LADs intranuclear spatial organization.

*The authors discussion of both the lack of correlation between fast/slow diffusion and heterochromatin and the increased autocorrelation for heterochromatin is helpful. It would be additional helpful evidence to support the biological meaning of the distinct diffusion domains (which otherwise can be criticized as the artificial cutoffs of an algorithm) if the authors could identify any biological feature that does correlate with the diffusion domains (since heterochromatin does not) they see?*

We do not have biological feature that does correlate with the diffusion domains, however, we could strengthen the hypothesis that heterochromatin domains are formed by a liquid phase-separation mechanism, by detection of a characteristic mobility reduction across phase boundaries.

**References**

1. Cho WK, Jayanth N, English BP, Inoue T, Andrews JO, Conway W, et al. RNA Polymerase II

cluster dynamics predict mRNA output in living cells. Elife. 2016;5.

2. Nagashima R, Hibino K, Ashwin SS, Babokhov M, Fujishiro S, Imai R, et al. Single nucleosome

imaging reveals loose genome chromatin networks via active RNA polymerase II. J Cell Biol. 2019;

3. Nozaki T, Imai R, Tanbo M, Nagashima R, Tamura S, Tani T. Dynamic Organization of Chromatin

Domains Revealed by Super-Resolution Live-Cell Imaging Article Dynamic Organization of Chromatin

Domains Revealed by Super-Resolution Live-Cell Imaging. Mol Cell. 2017;10:1–12.

4. Shaban HA, Barth R, Bystricky K. Formation of correlated chromatin domains at nanoscale dynamic

resolution during transcription. Nucleic Acids Res. 2018;46.

5. Chen CYA, Ezzeddine N, Shyu A Bin. Chapter 17 Messenger RNA Half-Life Measurements in

Mammalian Cells. Methods Enzymol. 2008.

6. Stasevich TJ, Hayashi-Takanaka Y, Sato Y, Maehara K, Ohkawa Y, Sakata-Sogawa K, et al.

Regulation of RNA polymerase II activation by histone acetylation in single living cells. Nature. 2014;

7. Senecal A, Munsky B, Proux F, Ly N, Braye FE, Zimmer C, et al. Transcription factors modulate c-

Fos transcriptional bursts. Cell Rep. 2014;8:75–83.

8. Kimura H, Sugaya K, Cook PR. The transcription cycle of RNA polymerase II in living cells. J Cell

Biol. 2002;

9. Wachsmuth M, Knoch TA, Rippe K. Dynamic properties of independent chromatin domains

measured by correlation spectroscopy in living cells. Epigenetics and Chromatin. 2016;9:1–20.

10. Strom AR, Emelyanov A V., Mir M, Fyodorov D V., Darzacq X, Karpen GH. Phase separation

drives heterochromatin domain formation. Nature. 2017;547:241–5.

11. Di Rienzo C, Gratton E, Beltram F, Cardarelli F. Fast spatiotemporal correlation spectroscopy to

determine protein lateral diffusion laws in live cell membranes. Proc Natl Acad Sci. 2013;

12. Peric-Hupkes D, Meuleman W, Pagie L, Bruggeman SWM, Solovei I, Brugman W, et al. Molecular

Maps of the Reorganization of Genome-Nuclear Lamina Interactions during Differentiation. Mol Cell.

2010;

13. Doi M, Edwards SF. The theory of polymer dynamics [Internet]. Clarendon, Oxford. 1986.

Available from:

http://books.google.fr/books?id=dMzGyWs3GKcC%5Cnhttp://www.catalog.enu.kz:55555/guid/A4A964

F6-C71A-4396-89B3-5F26CB3C232C.pdf

14. van Steensel B, Belmont AS. Lamina-Associated Domains: Links with Chromosome Architecture,

Heterochromatin, and Gene Repression. Cell. 2017;

15. Kind J, Pagie L, Ortabozkoyun H, Boyle S, De Vries SS, Janssen H, et al. Single-cell dynamics of

genome-nuclear lamina interactions. Cell. 2013;153:178–92.

16. Kind J, Pagie L, De Vries SS, Nahidiazar L, Dey SS, Bienko M, et al. Genome-wide Maps of

Nuclear Lamina Interactions in Single Human Cells. Cell. 2015;

**Second round**

**Reviewer 1**

The revised manuscript is significantly improved, but there is a key issue that remains unaddressed. The author presented a Hi-D method based on Bayesian interference to extract the Mean Square Displacement (MSD) from densely labeled chromatin. The calculation of MSD is currently obtained by SPT of the sparsely labeled loci. This approach, if proven to be correct, will be a nice addition to the existing methods for analyzing chromatin dynamics without the need to label single loci which is often not a trivial task. However, my major concern is that this method does not provide similar results as those with those with sparse labeling at single foci. An important validation is to compare the MSD extracted from sparsely labeled chromatin, with the results obtained with the Hi-D method presented here. The authors did this comparison only with simulation data, but not experimental data. The current experimental data the authors provided simply compared the fixed vs. live cells or quiescent vs. stimulated cells with and without serum, which are not direct validation of the method to SPT. This is a critical experiment to do. Without it, it is still not convincing that the MSD extracted from Hi-D is similar to that extracted from standard SPT.

An area that really concerns me is that the results presented in Fig. 4d and Fig. 4e appear to be inconsistent with a recently published work by Nagashima R et al. Single nucleosome imaging reveals loose genome chromatin networks via active RNA polymerase II. J Cell Biol. 2019;218(5):1511-30. This JCB paper used the standard SPT method with the sparse labeling of chromatin. They did essentially the same experiment of treating cells with DRB to inhibit transcription and found increased chromatin dynamics. They justified their conclusion from multiple experiments at various conditions, and the results are convincing. While Fig. 4d and Fig. 4e showed little change of chromatin dynamics after DRB treatment. No further experiments were conducted to address this inconsistency. Since Hi-D is a new method, it makes me wonder whether or not this Hi-C method generates the same conclusion as the standard SPT method. This is a critical issue that must be addressed to ensure future users who investigate biological problems that the inconsistent results are due to real biological phenomena not the method itself.

Therefore, given the lack of sufficient experimental validation, I still cannot support the publication of this paper.

**Reviewer 2**

In the revised version of the manuscript the authors have added more analysis. However, my main concerns have not been addressed. The manuscript remains very poorly written. There is hardly any experimental information, figures are poorly described which makes it very difficult to discern what is shown, experiments are not properly controlled, many figures rely on the analysis of one - single- cell. There is no mention of how many times experiments have been repeated and whether findings are consistent. Though I can appreciate the attractiveness of the method, the authors try to reach biological conclusions and for this, much more solid experimentation would be required. In addition the manuscript is almost impossible to follow, as several aspects are not properly explained. And this has not been improved by revision (on the contrary, it seems adding more analysis made the text even more cryptic and extrapolations rather bolder).

Just a couple of random examples (there are many, throughout the manuscript):

- figures 1, 2, 3 and 5 seem to all rely on the analysis of a single (the same in all figures!) cell, which was serum starved, imaged and then stimulated with serum and re-imaged. The details of the experimental procedure however are not described, , it has not been clearly mentioned that this one cell is shown in all 4 figures, it is not clear whether the cell in fig 1 and 2 is the cell before or after serum stimulation etc. It is not at all clear whether the experiment was ever repeated with similar results etc.

- When performing serum starvation/refeed (or in fact any physiological perturbation) it is essential to include controls showing that indeed cells are in G0 at the serum starvation step and will progress to S phase upon refeed (and when and to what percentage). This cannot be taken for granted, as slight variations in experimental design may affect the quality of the arrest and release. FACS analysis, or BrdU/EdU staining, and/or the use of cell cycle markers and/or markers of PolII activity can be used. When adding inhibitors controls are needed as well. Experimental procedures must be carefully described.

In its current form the manuscript remains in my opinion unsuitable for publication.

**Author appeal after rejection**

While I understand your decision to not publish our article as is, I wanted to formerly address the new issues brought up by reviewers #1 and #2[…]

In the case of reviewer #1, the major concern has been to compare my method to classic single particle tracking (SPT). This has been extensively addressed in figure 2 and the supplement. The reviewer dismissed this work since it used simulation data. However, I am comparing one analysis method to another it does not matter if the data is synthetic or not. The biological experiment the reviewer is now requesting, “sparse labeling at single foci”, is not possible to do at the same time as our imaging method mean it is simply not possible to compare them experimentally.

[…]The reviewer justifies not supporting publication of my article simply since we do reach the same conclusions as in Nagashima R et al. Single nucleosome imaging reveals loose genome chromatin networks via active RNA polymerase II. J Cell Biol. 2019. We also note that the method used in Nagashima et al., is far from the “standard SPT method” and has issues itself. We provide enough data to support our conclusions. It is not the aim of my article to deal with the inconsistencies of the recently published Nagahsima et al. 2019. Rather, I am using a new technique to study a few small biological perturbations.

Reviewer #2 starts with a statement saving the manuscript is very poorly written, which we believe is not true. The main issue raised by reviewer #2 is that “figures 1, 2, 3 and 5 seem to all rely on the analysis of a single (the same in all figures!)”. This is a gross misunderstanding the data. Yes, the images presented in those figures often do come from single cells but that is the point. They are not meant to be quantitative but representative. The quantitation is in figure 4. It would make no sense to generate a flow map from an average of cells. It would just look like a blurry mix. In figure 4 we show quantitation of parameters extracted from many single cells, thoroughly described in the figure legend with correct statistics.

The second point of reviewer #2 is that we do not control for serum starvation. Serum starvation is such a standard technique that this is a ridiculous ask. We grow our cells in preparation for microscopy. It is not possible to do FACS on cells in a microscope chamber. The reviewer alternatively suggests doing BrdU/EdU staining. Our analysis is in live cells which is incompatible with this. Even if we where to use something such as the FUCCI, live, cell cycle markers, our chromatin fluorophores are not present in these cell lines and would not be compatible. In the end, we know that our cells are in G0 from serum starvation simply because they stop dividing in the microscope chamber.

[…]

I believe my method is a significant advance over what I published previously in NAR. Using this method, it is now possible to tell the types of motion throughout the whole the nucleus i.e. directed motion, confined etc rather than at discreet loci. In addition, this method is far easier to implement than that presented in Nagahsima et al. 2019 since does not require photoactivation. Finally, I also note that the chromatin dynamics field is excited about this work with the bioRxiv version of this work already having been cited: Advances Using Single-Particle Trajectories to Reconstruct Chromatin Organization and Dynamics, Trends in Genetics, 2019.

**Reviewer 3 comment on author appeal**

Like Referee 1, I am interested to know the reason behind differences between Hi-D and SPT. And how to reconcile their claim that heterochromatin and euchromatin don’t experience different dynamics with a recent BioRxiv paper describing the fact that heterochromatin interactions are more stable (fall apart slower under digestion) than euchromatin. But, ultimately, different results from an orthogonal method is not a reason to prevent publication. As long as the authors have done as much as they can to properly control and present their own results, these differences should be out there in public record to be further debated. Deeper biological insights may come from identifying why Hi-D and SPT appear to give different results, but that may be beyond the current paper. The addition of the simulation is nice. The problem would be if there are things going on biologically that are not accounted for in the simulation, then comparing methods in the simulation wouldn’t capture the whole situation. But, at least, it allows the demonstration of how the methods would compare in a certain scenario. (I have to admit, understanding what they are presenting as results from simulation was not quickly easy to grasp and I might need to stare at it for longer before I can really understand what they are trying to claim from this—if you feel you need additional close scrutiny of whether their simulation is correctly executed, interpreted, and presented, I’ll need to examine it longer). I don’t agree with the authors that what the reviewers require would be SPT and HiD in the same cells at the same time. A parallel SPT experiment in the same condition would be sufficient comparison in my mind. If you take these cells in this same condition and monitor heterochromatin and euchromatin spots with SPT, can you see the same results? But, I can also see that maybe this is requiring too much.

I do not think that the [Referee 2’s] request is ridiculous. Yes, serum starvation is common, but it is also commonly cause for concern (I hear others, particularly very physiologically oriented colleagues, speak about this). These are cancer cells (which may not respond like all other previously used cells in serum starvation) and serum starvation is a whole biological response, not just targeted transcription inhibition. Replication and cell cycle phase affect chromatin structure and dynamics a lot, so even if it’s “unlikely” that this is changing very much, it’s not really reasonable to dismiss this as an unfair criticism. Major and somewhat surprising (chromatin motion slows down when ATP driven activities increase! Heterochromatin and euchromatin don’t move differently!) conclusions are made based on serum starvation and release. I think it’s fair for the community to ask for carefully described and controlled experiments to underlie this. And, the referee may sound like they are asking for a lot, but a simple flow cytometric analysis on a sample batch of cells under the same conditions can be completed in one day with very basic flow cytometers (not requiring cell sorting as implied by the referee’s “FACS” comment-- I expect that was just a typo by the reviewer). “Seeing the cells stop dividing” is not convincing: justifying things by this method would require some kind of quantitative imaging or counting of cell numbers in the dish over time, which is actually much harder to do than flow cytometry or BrdU staining. And they don’t have to do BrdU staining in live cells to address the concern-- just serum starve and refeed as usual and at the timepoint where measurements are typically taken, then fix and stain. I get the impression that the authors feel attacked (perhaps because all of us are concerned about whether this is “novel enough”, which is always an irritating comment to an author) and therefore are interpreting reasonable requests as unreasonable and petty. But when you are working with cancer cells in culture (easy to grow) and doing a perturbation that takes a few hours, saying it’s not possible to use flow cytometry or BrdU to measure cell cycle state is pretty unreasonable. Even if they feel this has been done before, it hasn’t been done in identical conditions to their experiment in their lab-- they should just do it to quell any concerns.

I think my overall response is: the things the authors have added are helpful (simulations, etc.). The authors should not be held up from publishing forever just because their method doesn’t align precisely with previous publications; that kind of thing can be debated in the field for years to come. But, the referee concerns asking for thorough experimental explanations and controls are not unreasonable and should not be hard for the authors to comply with.

And, finally, I looked at their response to my statement: “I would appreciate a more explicit description of how the approach to diffusion classification taken here revealed insights that were not apparent from their earlier description of velocity and correlated motion.” Their response, as I understand it, is essentially “Don’t worry, raw motion gives the same results as diffusion constant calculation”. I guess that’s comforting in reassuring that their conclusion is not wrong, but it doesn’t really show me why their diffusion constant calculation is insightful. If raw motion (which is much more straightforward to calculate, understand, and doesn’t involve employing various physics assumptions which could be debatable) gives the same results, why do we need diffusion constant and anomalous diffusion estimation?

**Authors’ response**

To provide a control for cell cycle stage in the serum starvation experiment, addition of serum for 15 min to previously starved cells increases mRNA levels to 50% of the levels measured in exponentially growing cells. We confirmed that addition of serum to serum starved U2OS cells activates transcription and increases the proportion of cells in G1 (Supplementary Figure 10). Accordingly, we added the following sentence to the main text. An explanation of the FACS experiment in the method section and supplementary figure show the results.

Transcription is largely inhibited in cells grown in a serum-free medium (Supplementary Figure 10).

We first comment on Reviewer #3’s question “[…] why do we need diffusion constant and anomalous diffusion estimation?”.

In the original submission of the manuscript, we were only looking at the distribution of diffusion constant and anomalous exponent in eu-/heterochromatin. We then agreed with Reviewer #3 that the conclusion reached by this analysis may be compromised by the downstream motion classification by the Bayesian inference and that ‘raw’ motion would give a more straight-forward analysis, which we added after the first revision. The conclusion reached by either inferred biophysical parameters and ‘raw’ motion agreed, reassuring us in our conclusion. However, agreement between inferred parameters and raw motion does not mean that the parameter inference (and associated motion classification) is redundant. Raw motion displays (only) stochastic motion while inferred parameters display time-average and, importantly, deterministic physical quantities. These quantities are much more useful than stochastic motion as they allow understanding and capturing the physics of chromatin dynamics, while raw motion only displays subsequent snapshots of the underlying physics. The inference scheme is thus of great importance to the analysis of global chromatin dynamics.

To relate the Hi-D method to our previously published DFCC, let us first emphasize that both Hi-D and DFCC build upon the reconstruction of flow fields of chromatin (by means of Optical Flow). Then, DFCC spatially and temporally correlates the flow field in order to quantify the extent to which correlated motion is present in various biological conditions. The result is a spatially averaged correlation curve which is further quantified in order to yield the correlation length and the smoothness parameter. Hi-D in contrast does not compute correlations from flow fields. Hi-D aims to compute spatially resolved biophysical parameters deterministically describing the diffusion of chromatin. This means that Hi-D assigns parameters to each pixel (n= >10000 per nucleus) of the image series while DFCC assigns averaged parameters to the entire nucleus (n=1).

The methods thus greatly differ by means of the downstream analysis of flow fields. Raw flow fields are, in general, of little impact since those depict stochastic motion. The inference of quantities of interest (extent of correlation in the case of DFCC and parameters describing the diffusion process in the case of Hi-D) is the most important parts in the analysis of chromatin motion based on flow fields. Significantly, the biophysical parameters deduced from Hi-D are to get the root of mechanisms drive the chromatin organization. In order clearly state the advances of Hi-D over DFCC, we modified the introduction of the article:

A first study analyzing bulk chromatin motion at nanoscale resolution revealed dynamic partitioning of chromatin into a number of nuclear sub-regions which show correlated motion of chromatin in the micrometer range [20]. However, the cause and/or effect of (correlated) chromatin dynamics is not yet clear. Likewise, whether compaction of chromatin determines its spatial coherence or whether chromatin dynamics are distinct in open and closed chromatin is still a matter of debate.

To tackle this need, we developed a new approach called High-resolution Diffusion mapping (Hi-D). Hi-D is a method overcomes the limitations of sparse and ensemble approaches. Hi-D combines dense optical flow reconstruction to first quantify the local motion of chromatin at sub-pixel accuracy from a series of images, and a Bayesian inference approach in the second step to precisely classify local types of diffusion. Biophysical properties such as diffusion constants and anomalous exponents are determined for each pixel of each image in the series to create two dimensional maps of chromatin dynamics at single pixel resolution in living single cells.

We appreciate the suggestion of an experiment comparing SPT and Hi-D. However, in our opinion a comparison between the two methods is not reliable and thus not valuable if the SPT spot and the surrounding chromatin cannot be imaged in two colors in the same cell. To see why, consider the extensive heterogeneity in recorded dynamics of any labeled genomic locus in vivo. Each cell behaves differently from the others and locus dynamics greatly depend on experimentally uncontrollable factors such as the locus positioning. Furthermore, chromatin movements are foremost stochastic such that the exact movement of a given locus at a given time can, in general, not be predicted with certainty from the past displacements. In turn, both SPT and Hi-D aim to reconstruct the stochasticity of the locus motion between two subsequent frames of a recorded series, ideally yielding the same displacement vector. This is not possible if the SPT spot and the surrounding chromatin are not imaged in the same cell and at the same instantaneous time (for instance the channels are imaged in alternating frames). Unfortunately, we do not have access to a suitable microscope to perform such an experiment (2-channel simultaneous imaging of SPT spot and chromatin in single cells).

Furthermore, and more importantly, we agree that the comparison of Hi-D to SPT appears obvious to the community. However, we must insist that both methods tackle drastically different imaging conditions, which is the major reason why such a comparison is tricky/impossible. We included the explanation of this issue in the Supplementary Material (new Supplementary Note 4 and Supplementary Movie 1) to make it clear to the reader why one should refrain from a direct comparison between SPT and Hi-D. We also briefly mentioned this issue in the main text, referring to Supplementary Note 4 and Supplementary Movie 1 for further explanation:

One should, refrain from a direct comparison between dynamics inferred by SPT and Hi-D, because the two methods are aimed at analyzing images from drastically different conditions (Supplementary Note 4; Supplementary Movie 1).

**Third round**

**Reviewer 3**

The authors have done good careful work to address the concerns that the reviewers expressed. With the additional controls (such as flow cytometry and re-introduction of serum), readers will have what they need to understand and evaluate the results. The additional explanations of the purpose and difference between their method and other approaches is helpful. I am satisfied with the revisions at this point.
